# Web-Based Self-Management Programs for Bipolar Disorder: Insights From the Online, Recovery-Oriented Bipolar Individualised Tool Project

**DOI:** 10.2196/11160

**Published:** 2018-10-24

**Authors:** Kathryn Fletcher, Fiona Foley, Greg Murray

**Affiliations:** 1 Centre for Mental Health Faculty of Health, Arts and Design Swinburne University of Technology Melbourne Australia

**Keywords:** Web-based intervention, bipolar disorder, self-management

## Abstract

**Background:**

Bipolar disorder (BD) is a complex, relapsing mood disorder characterized by considerable morbidity and mortality. Web-based self-management interventions provide marked opportunities for several chronic mental health conditions. However, Web-based self-management programs targeting BD are underrepresented compared with programs targeting other psychiatric conditions.

**Objective:**

This paper aims at facilitating future research in the area of self-management of BD and draws insights from the development of one such intervention—the Online, Recovery-Oriented Bipolar Individualised Tool (ORBIT)—that is aimed at improving the quality of life of people with BD.

**Methods:**

We have discussed the opportunities and challenges in developing an engaging, evidence-based, safe intervention within the context of the following three nested domains: (1) intervention development; (2) scientific testing of the intervention; and (3) ethical framework including risk management.

**Results:**

We gained the following insights across the three abovementioned overlapping domains: Web-based interventions can be optimized through (1) codesign with consumers with lived experience to ensure relevance and appropriateness to the target audience; (2) novel content development processes that iteratively combine evidence-based information with lived experience perspectives, capitalizing on multimedia (eg, videos) that the digital health space provides; and (3) incorporating Web-based communities to connect end users and promote constructive engagement by access to a Web-based coach.

**Conclusions:**

Self-management is effective in BD, even for those on the more severe end of the spectrum. While there are challenges to be aware of, guided self-management programs, such as those offered by the ORBIT project, which are specifically developed for Web-based delivery provide highly accessible, engaging, and potentially provocative treatments for chronically ill populations who may otherwise have never engaged with treatment. Key questions about engagement, effectiveness, and cost-effectiveness will be answered by the ORBIT project over the next 18 months.

## Introduction

### Background

The well-documented strengths of Web-based delivery for psychological interventions—flexible access across space and time, low cost, and potential for personalization [1-4]—have been bolstered with growing evidence that clinical effect sizes for many common clinical presentations are as large as those achieved by traditional face-to-face interventions [5-7]. As more and more of human life is mediated through technology, it is, perhaps, not surprising that the archetypal personal encounter of psychotherapy is also finding its feet on the Web.

The aim of this paper is to accelerate progress toward the next generation of Web-based interventions by critically reviewing the experience of one, very particular, Web-based treatment development process. The overarching aim of the Online, Recovery-Oriented Bipolar Individualised Tool (ORBIT) project was to develop and test a Web-based self-management intervention for people with bipolar disorder (BD) to improve their quality of life [8,9].

BD is a complex, relapsing mood disorder characterized by considerable morbidity and mortality. Functioning levels vary widely between and within individuals with BD, presenting a challenge for services organized primarily around management of chronically low-functioning patients [10,11]. Web-based self-management programs for this group represent a unique opportunity to address this need [12]. Unfortunately, Web-based self-management programs targeting BD are underrepresented relative to programs targeting other psychiatric conditions [13]. Developing an engaging, evidence-based, safe Web-based self-management program for individuals with BD presents not only special challenges but also opportunities.

**Figure 1:**
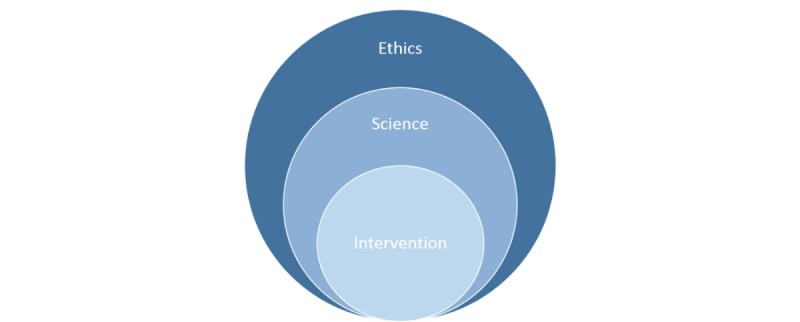
Project domains.

We hope that the information presented here will be of use to others developing and testing Web-based psychological interventions. While we focus on a Web-based intervention for BD as a guiding example, our learnings are generalizable to interventions for other mental health conditions. Insights are consequently organized in the following three domains: (1) development of the intervention; (2) development and conduct of a rigorous scientific test of the intervention’s efficacy and mechanisms of action; and (3) development of a best-practice risk management and ethical framework for the trial (and ultimately for the roll-out of the intervention). From an overarching project perspective, it is useful to think about these three domains as nested (Figure 1).

### The Online, Recovery-Oriented Bipolar Individualised Tool Project

Our international team is currently in the latter phases of recruitment for the randomized controlled trial (RCT) component of the ORBIT project. The trial (registered 23 June, 2017, ClinicalTrials.gov: NCT03197974) is funded by the Australian National Health and Medical Research Council and was reviewed and approved by Swinburne University of Technology Human Research Ethics Committee (2016/289). As detailed in the protocol paper [9], recruitment is primarily via open social media sites (eg, International Bipolar Foundation Facebook site). Main inclusion criteria are as follows: a diagnosis of BD (confirmed via a phone-administered structured diagnostic interview), no current mood episode, history of ≥10 mood episodes, no current psychotic features or active suicidality, and under the care of a medical practitioner. The trial compares two contrasting interventions, both referred to as self-management programs (Mindfulness vs Psychoeducation) for BD [9]. The Web-based programs aim to improve the quality of life in those with “late stage” BD (defined as ≥10 mood episodes). The programs (accessible via personal computer, tablet, and mobile phone) are brief (4 modules delivered over 5 weeks), self-paced and tailored to BD. They incorporate a range of multimedia components to maximize engagement and motivation: videos of consumers with the lived experience and clinicians (shaping program content), audio files for practicing learned concepts, interactive exercises, quizzes, static images, and downloadable PDF content for further learning opportunities. Guided support is offered via once-weekly asynchronous messages (from trained coaches); peer support is offered via moderated forums and the ability for users to connect privately with each other via a secure-messaging system embedded in the program.

The programs are intended to be highly interactive; users are encouraged to track and monitor well-being via an embedded tracking tool, complete interactive exercises and reflect on their participation as they complete each module, contribute to forums, connect with other users (fostering social support), and message their coach for assistance. Furthermore, engagement and adherence are encouraged via coach messages, seeded forum posts, and cognitive behavioral principles to facilitate the practice of skills in everyday life.

Unlike the standard approach of adapting validated face-to-face psychological interventions for Web-based delivery, the programs were bespoke Web-based interventions. Content was driven by evidence-based psychological treatments, offered via the following two arms: (1) Psychoeducation for BD (serving as the active control condition) and (2) Mindfulness for BD (incorporating elements of mindfulness-based cognitive behavioral therapy, self-compassion, and Acceptance and Commitment Therapy). Full details of the ORBIT project, including the rationale for targeting “late stage” BD are outlined in the protocol paper [9].

Insights from the ORBIT project are now considered in terms of strategies to optimize the intervention, scientific considerations for testing the intervention, and the framework that was developed to optimize ethical conduct of the research and delivery of the intervention.

## Key Learnings: Opportunities and Challenges

### Intervention

Here the intervention offered in the ORBIT project refers to the Web-based self-management programs. A number of strategies were used to optimize these programs, now detailed.

#### Consumer Input

Consumers with lived experience of BD were involved in all phases of program development, aligning with the consumer-based participatory research approach [14]. Consumer feedback from the pilot phase [8] guided content development for the current iteration of ORBIT. Intervention content is largely driven by the consumer voice, captured via videos of those with lived experience of BD. A local consumer advisory group (CAG) was established for the ORBIT project, comprising 10 individuals (6 females and 4 males) diagnosed with BD. Three CAG meetings took place during the development phase. Members played an integral role in providing feedback on content and the website itself (look-and-feel, ease of use, any perceived benefits or barriers) to ensure appropriateness and relevance to those with BD. We additionally consulted our pre-established CAG (located in Canada), part of the Collaborative Research Team to study psychosocial issues in BD (CREST.BD), to ensure that intervention content was appropriate for a broader international audience.

As part of the RCT, qualitative feedback from participants completing the program was collected to (1) guide future developments and (2) provide insights into their level of engagement with the Web-based intervention. These practices extend beyond the usual application of face-to-face intervention for the Web-based format by allowing a largely consumer-driven process. This approach ensures the program is tailored to the population it seeks to serve and aligns with the recovery model focus of empowering consumers via their active involvement in all stages of intervention development and testing.

#### Use of Multimedia to Develop Content and Explain Concepts

Our international team of researchers, clinicians, and consumers initially developed and piloted program content drawn from mindfulness-based therapies [8]. The program was then extensively revised and extended, iteratively developed via a dynamic interplay of theoretically derived therapeutic content and footage from videos of consumers with lived experience of BD. Content for Web-based interventions is often adapted from a face-to-face psychological intervention for Web-based delivery, as part of a one-step process where the content is largely defined upfront. By contrast, ORBIT content was progressively developed over a 6-month period. The process commenced with the “top-down” development of a semistructured interview schedule, drawing from broad topic areas aligned with mindfulness-based therapeutic approaches and BD psychoeducation. Clinical psychologists and mindfulness practitioners were consulted on topic areas to ensure that questions were grounded in a psychological framework. Next, 12 consumers were recruited to participate in the filming process: consumers were selected on the basis of gender; age; cultural background; and a range of experiences with mindfulness, acceptance-based approaches and self-management strategies (eg, recognizing early warning signs and triggers), ensuring a representative sample that ORBIT participants could relate to. The video process adopted a documentary-style interview, lasting up to 2 hours per person, undertaken with a professional film crew. Consumers were encouraged to speak from personal experience to ensure footage captured the central “consumer voice.” This “bottom-up” generation of program content was balanced within the “top-down” psychological framework—footage was carefully reviewed, and the project team iteratively revisited the planned content and structure of the intervention to maintain this balance. Hours of footage were edited into “snippets” from a range of consumers and combined into short videos (3 minutes on average in length), describing particular skills and experiences. This new type of delivery (documentary-style videos as an engagement strategy for Web-based interventions) ultimately led to a novel way of developing a Web-based intervention and, ultimately, a new intervention. The final stage of development involved fleshing out content from videos, including key summary messages, supplementary text, and MP3 audio files (allowing for in-the-moment experiential practice of newly introduced concepts) to promote learning and encourage skill development. Program content was then reviewed by our local and international CAGs and revised on the basis of their feedback. Overall, feedback indicated that videos successfully captured the consumer voice, providing a “real-world” feel that people could relate to, adding credibility to the program (balancing realistic messages with hope), reducing stigma, and allowing subtle processes to be described in an engaging and personalized way:

I like the real experiences shared by those with Bipolar better than those put forward by clinicians. A shared personal lived experience of Bipolar resonates deeply. Whereas I always feel a clinician is regurgitating something from a textbook or re-telling someone else’s personal story: they haven’t lived it, so they will never truly know.

Female, age 51 years, bipolar II disorder

CAG members were consulted on the duration of videos; in general, there was agreement that “shorter” (3-4 minutes) videos were preferable in terms of maintaining interest and engagement, without being too cognitively demanding. This is in accordance with our prior experience in developing video-based content for Web-based interventions for individuals with persisting psychosis as a strategy to promote recovery [15]. Finally, the use of informational PDFs, clinician videos, and instructional videos (eg, consumer or expert walk-through, how to use a mood tracking tool) allows concepts to be easily understood within a user-friendly context. The Web-based environment lends itself to participants revisiting content as needed, at their own pace, to consolidate their understanding and facilitate repeated practice.

Content is guided by psychological principles; thus, consideration of how this can be made engaging, interactive, flexible, and appealing is essential to the success of any Web-based self-management program. Given the cognitive impairments common in those with BD, practical considerations (eg, being able to start, pause, and recommence topic areas; videos of short duration accompanied by transcripts; length of exercises; language; and neutral colors and icons) were carefully considered to ensure ongoing engagement.

#### Access to a Web-Based Community

The programs were designed to maximize constructive engagement by participants having access to a Web-based community (other participants, moderated forums), allowing them to link in with social support that may not otherwise have been realized [16]. This can provide a sense of normalization and a way in which they can also support others. The forum facilitates sharing of key learnings as participants navigate through the program, and a secure-messaging system allows participants to foster connections with other users if they so wish (extending social support networks outside of ORBIT). Participants are assigned a Web-based coach as part of the ORBIT community, with asynchronous message support. As overviewed by others [17], support in Web-based interventions for BD and other psychological conditions reduces dropout rates, making them comparable to rates observed in face-to-face therapies. Indeed, motivation to persist with Web-based interventions has been found when needs for relatedness (eg, identifying with other end users and content, support from Web-based coach, forum participation) are satisfied [18].

#### Clinical Cautions

**Figure 2:**
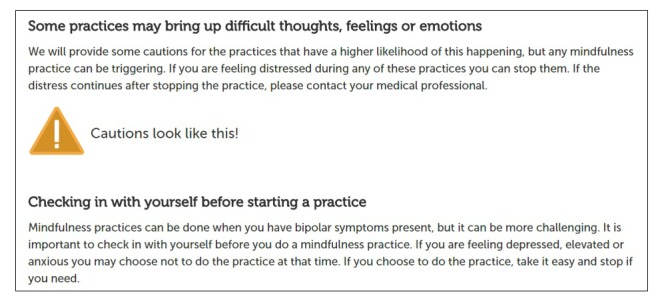
Clinical cautions. Source: the ORBIT program.

Our group has been interested in developing novel psychotherapies for BD, drawing on third-wave principles of mindfulness and self-compassion [19]. There have been anecdotal concerns in the literature about potential iatrogenic effects of mindfulness for people with BD [8]. One of the arms in the ORBIT project contains such elements, requiring meticulous attention to ethical issues and risk management. Current mood state is an important factor to consider when introducing those with BD to experiential techniques within a Web-based intervention, given the risk of triggering mood dysregulation. Clinical caution messages (both within the content and incorporated into audio exercises) are used to empower participants to consider whether practicing a particular technique at that moment would be beneficial or should be delayed. For instance, anecdotal reports of body scan exercises that are lengthy (eg, 20 minutes or longer) indicate they can be triggering (of mood episodes) for some; thus, cautions based on current mood state can be particularly useful (Figure 2).

This type of encouragement allows participants to self-pace through the program, allowing for increased engagement and a sense of autonomy. Participants are encouraged to reflect on exercises, and any adverse events arising that are directly related to intervention content are logged.

#### Sustainability

A widely recognized problem in internet delivery is transitioning from research-funded development and trialing of an intervention to long-term sustainable delivery outside a research funding environment. Swinburne University of Technology has been at the forefront of improving the sustainability of Web-based interventions by advocating (successfully) for the national recognition of eTherapy hours as integral to the training of Clinical Psychology Masters students (25 eTherapy hours approved by the Australian Psychological Society College of Clinical Psychologists). In the ORBIT trial, and in future potential iterations of the ORBIT website beyond the research phase, the primary personnel support (coaching) is provided by Clinical Psychology Masters students as part of their first internal placement at the university, a model that can be generalized to any psychological interventions offered in Australia.

### Science

#### Randomization and Allocation

A key facet of minimizing bias in treatment trials is ensuring that participants are randomly allocated to comparison conditions [20]. Web-based RCTs benefit from the use of fully automated computer-generated blocked randomization *and* allocation methods, ensuring all aspects of randomization are fully concealed to research personnel. Such methods are superior to traditional RCT methods (eg, sequentially numbered opaque sealed envelopes, central randomization by telephone to a trials office, etc) as no human involvement is required (an airtight method of concealment, reducing bias) and time efficiency is achieved (randomization and allocation can occur within seconds of each other, allowing participants to commence the intervention almost immediately). The latter is particularly important for studies involving those with frequent mood changes (ie, BD) that can occur within hours or days, impacting study data.

#### Defining “Dose” in the Web-Based Context

A central challenge posed by the Web-based context is how best to define intervention “dose.” An effective “dose” can constitute the level of usage needed for participants to benefit from the use of the program, the extent that the “dose” varies between participants, and participant characteristics that may influence the “dose” that is needed [21].

Participants differ in Web-based usage patterns, with evidence suggesting that less time will be spent on the program than researchers expect [22]. Program usage statistics automatically recorded onsite (eg, number of pages viewed, number of tasks completed, timestamps, etc) are an essential measure of adherence and engagement, providing an objective proxy for “dose” received. Study investigators need to identify program usage statistics of interest during the development stage to communicate this to the website developer (who will incorporate selected variables into data downloads for later analysis). Usage data can automatically be tracked and operationalized in an algorithm combining time on the Web, activity completion, and active engagements with the intervention [23]. However, adherence (and the related construct of engagement) is difficult to characterize and measure—for instance, the proportion of time spent on a particular page does not necessary represent the time participants were actively engaged on that page (they may have been chatting on Facebook or away from their computer). Furthermore, adherence alone does not capture a participant’s entire experience of an intervention [23]; there is growing interest in understanding other ways in which individuals *engage* with Web-based interventions, and the meaning of *engagement* in this context [24]. Currently, minimal consensus exists on the definition and conceptualization of engagement with Web-based interventions. Some view engagement as synonymous with adherence and the opposite of “intervention attrition” or “treatment dropout” [25,26], while others consider it to move beyond mere attendance, incorporating the extent that an individual *actively* participates in a treatment on offer behaviorally, cognitively, and affectively [27,28].

To develop a more comprehensive understanding of adherence and engagement, the ORBIT project will examine both objective and subjective levels of “active” participation; usage statistics will be captured via the website, and in-depth qualitative interviews will be conducted with a subset of participants asking about their usage (online and “offline” practice of skills) and *perceived level of engagement* with the content. Through this process, we will develop an innovative algorithm to quantify the important variables of “minimum dose” and “attrition” in the Web-based context for inclusion in statistical analyses. As discussed in the protocol paper [9], the concept of attrition appears in two sensitivity analyses of the primary outcome. First, per-protocol analyses will be conducted on those receiving a minimal dose of the intervention, with minimal dose to be defined on the basis of the pending algorithm of self-reported and automatically recorded usage variables. Second, intention-to-treat analyses will be repeated with imputation on baseline and relevant postrandomization variables; these variables could include attrition (again defined in the pending algorithm of usage variables) if it is shown to differ between groups. As also outlined in the protocol paper, we will attempt to follow and assess all participants regardless of the level of usage of the site during the intervention period (with the exception of those explicitly discontinuing or being withdrawn on ethical grounds).

#### Minimizing Nonadherence and Maximizing Engagement

As outlined in our protocol paper [9], the interventions follow best practice in persuasive system design. Three key features known to impact on engagement are utilized: (1) *dialogue support* (praise from coach and forum moderator, email reminders); (2) *social support* (social facilitation through discussion threads in moderated forums); and (3) *primary task support* (best-practice principles for modularization of content, personalization or monitoring of progress, prompted self-monitoring, and rehearsal) [29]. Furthermore, the intervention is brief, and program content is released to each participant sequentially each week in an attempt to pace users as they work their way through the program, while gradually building on knowledge from earlier weeks (with “teaser” messages with respect to upcoming content). We opted against the delivery strategy of a single exposure (receipt of all content in one go), despite some evidence suggesting engagement rates increase when end users have control over how they view content, along with free choice on when they interact with it [30]. Our decision was guided by qualitative consumer feedback from our pilot study [8], indicating some end users felt overwhelmed with the amount of content.

**Figure 3:**
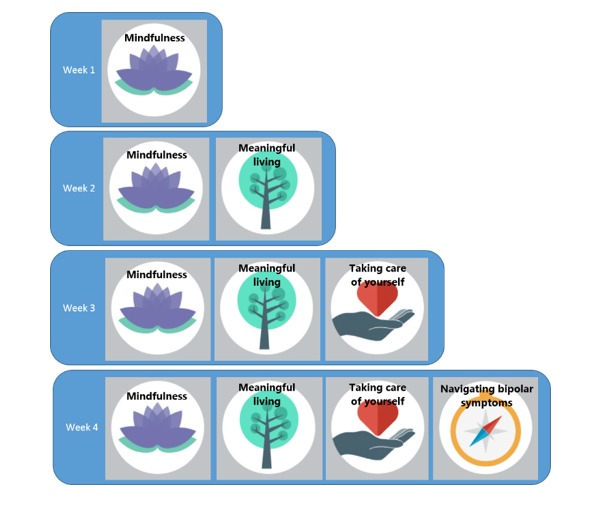
Online, Recovery-Oriented Bipolar Individualised Tool (ORBIT) topics. Source: the ORBIT program.

An open approach to navigation within each week’s content invites a further challenge—the program is essentially a “set of offerings” rather than “sessions” or “modules” to be completed, catering where possible for different users (eg, those with no experience of mindfulness vs regular meditators; those who have limited time to spend on the program). Unlike manualized treatment programs adapted for the Web-based format, one cannot assume that participants will work their way through each week’s content in a sequential manner. This required careful consideration during ORBIT content development, with persuasive technologies (eg, links and “suggestions” embedded within the content) used to prompt participants on how they might best navigate their way through the program, “chunking” of material to allow for shorter sessions times depending on user preferences, and suggestions for skills practice peppered throughout the content in case users did not click through to the “homework” page located at the end of each week’s content. Topic areas were crafted in a largely self-contained way, for example, using mindfulness (ranging from beginners exercises to more advanced practices) as a guiding overall skill to integrate topics (Figure 3).

#### Consumer Involvement

Consumers (referred to as “superusers”) with lived experience of BD were employed for the ORBIT project and trained to assist with seeding forum content and facilitating engagement with the content. This strategy brings unique opportunities and challenges. While a rich and lively forum community has developed and participants appreciate and benefit from communication with peers, guiding principles have been developed to manage the ethical and scientific impact of this dynamic environment. The superuser role is defined as nontherapeutic, and a degree of self-disclosure is encouraged.

From a scientific perspective, superusers are instructed to seed forum content with messages that are specific to the program being offered (eg, mindfulness-related material), avoiding (where possible) cross-contamination with content from the alternate program. Monthly supervision of superusers is undertaken to ensure that these guiding principles are adhered to while balancing the tension of allowing the forums to organically unfold as part of a dynamic Web-based community.

### Ethical Framework

Remote delivery considerations associated with Web-based self-management programs include risk-management, participant distress, and legal issues around delivering interventions in different jurisdictions.

#### Risk Management

Delivering an adjunctive Web-based self-management program for BD (and indeed, any chronic mental health condition with substantial clinical risks) within the context of an international research trial brings a level of risk that requires clear protocols should adverse events arise. Real-time intervention is not always possible or feasible given users are on the Web during different time zones, and its use must be carefully considered.

Motivated by clinical risk-management priorities, as well as principles around generalization of learning through participation, an approach was developed that emphasizes participants’ local networks of treatment and care [31]. Specifically, our approach was to explicitly devolve safety and well-being to participants and their local network (treating mental health practitioner and local emergency services). This is achieved in three key ways. First, an inclusion criterion requires participants to have had contact with a mental health professional during the past 12 months, to provide these details to the project team, agree for this professional to be contacted if necessary, and understand that they remain the first point of contact. Mental health professionals are posted a courtesy letter informing them of their clients’ participation in the ORBIT project. Second, participants are explicitly made aware (via the consent form, during the sign-up process when speaking to the research team, information on the program websites, coach messages, and forum messages from superusers) that the program is not intended to replace their usual care, does not provide a crisis service, and is not monitored in real-time. Links to emergency resources are, nonetheless, provided on the program websites (eg, *unsuicide.wikispaces.com*). Participants are directed back to their mental health professional as needed throughout their involvement in the ORBIT project. Third, a “red flag” protocol was developed to guide adverse event procedures, based on our experience with other Web-based interventions and websites for BD [32-37] and consultation with the *CREST.BD* CAG. This is detailed in the protocol paper [9]. In essence, the decision tree distinguishes between red flag information (which can arise during research assessments, Web-based questionnaires, forum posts, and messages to coach), suggesting immediate risk of harm for which real-time intervention is feasible or recommended (eg, when active suicidality is identified during a phone assessment), and when it is not (eg, when the research team becomes aware of active suicidality mentioned in a forum post from 48 hours previous). Actions are progressively escalated to senior staff members if required. As an example, forums are moderated by the project manager; inappropriate content is initially flagged (both in terms of risk and potential for triggering other participants) by superusers, prompting appropriate actions. All trial staff are comprehensively trained on these protocol procedures, operating to the guiding principle that participants’ local treatment and care networks are not disrupted. As per standard ethical guidelines, participants are withdrawn from the ORBIT project on a case-by-case basis should it be deemed that their well-being is compromised by their participation, and serious adverse events suspected or known to be related to participation in the trial are reported to the local administering ethics committee (Swinburne University of Technology Human Research Ethics Committee).

#### Participant Distress

Related to risk management, participant distress (whether arising as a direct result of participation in the project or as part of the usual clinical course of the mental health condition) is an ongoing challenge and particularly so for remotely delivered interventions. As for the majority of RCTs (whether Web-based or face-to-face), the ORBIT project includes a structured diagnostic interview, the Mini-International Neuropsychiatric Interview [38] to assess inclusion or exclusion criteria. Structured diagnostic interviews are, by their very nature, highly detailed and require participants to revisit their experiences of distressing symptoms. When conducted face-to-face, interviewers are able to pay attention to nonverbal cues and can manage distress levels sensitively. This poses a particular challenge for interviews conducted over the phone, as for the ORBIT project. Distress associated with participation in the diagnostic interview has been flagged by consumers participating in training for research staff administering the Mini-International Neuropsychiatric Interview, as well as a small proportion of ORBIT project participants. The detailed nature and duration of the baseline interview (lasting up to 2 hours) can be particularly distressing for those who have experienced multiple mood episodes. Solutions to date have included warning participants upfront of the potential for distress arising, interviewers being trained to tune in to participant tone of voice and other verbal cues, offering frequent breaks during the interview and conducting the interview across a few sessions if needed. While structured diagnostic interviews are a standard component of mental health research, sensitivity to participant distress and burden (particularly for those with chronic disorders such as BD) is a key priority. Offering participants the opportunity to debrief following such interviews may be an additional solution for future studies in this space.

#### Legal Issues

A key issue currently facing the delivery of Web-based “interventions” for mental health concerns the type of intervention that is offered. Interventions claiming to have some therapeutic value (eg, psychological interventions) fall into a gray legal zone, whereby certain jurisdictions require the “therapist” providing the “intervention” to be registered in the state, territory, or country where the client accesses the service from. Indeed, some states could potentially prosecute the remote “therapist” under the state’s laws. As clients can access Web-based programs from any location worldwide, this presents a legal minefield. As a first step to navigate this, the terminology used to describe the intervention must be carefully specified—for example, the ORBIT project does not claim to offer a psychological service, rather a self-management program that complements (but does not replace) usual clinical care. Second, care should be taken in defining what the program is intended to offer—the ORBIT project indicates that improvements in quality of life may result from completing the program. Third, as a guided program where participants have access to a personal coach, coach qualifications and role are made clear upfront. Specifically, the coaching role is nontherapeutic, with the key aim of supporting participants in terms of their *engagement with program content*. The tone and content of coach messages are carefully crafted on the basis of general guiding principles to standardize responses where possible (while remaining “human”), and coaches receive regular supervision. For example, responses tending toward therapeutic statements (eg, advanced empathic insights) were discouraged on two grounds: (1) the asynchronous email communication cannot sustain such a dialogue and (2) these could weaken participants’ engagement with their own local therapeutic resources. Finally, as a Web-based program within the context of an RCT, we strategically positioned the ORBIT project as a “single-site” study, governed by a single Human Research Ethics Committee (HREC; in this case, Swinburne HREC), offering services delivered from Australia outlined in a disclaimer present in the website terms and conditions reading, “*The services on the ORBIT website are provided in accordance with Australian laws and health practice standards. You acknowledge and accept that the services may not comply with the laws and standards that apply in the jurisdiction in which you receive the services.* ” Many of the legal issues associated with the delivery of Web-based interventions remain unknown at present while university HRECs have not developed internal processes to deal with such issues.

#### Summary of Learnings

Insights across the three overlapping domains (intervention development, scientific testing, and ethical frameworks) are now summarized. Web-based interventions can be optimized through (1) codesign with consumers with lived experience to ensure relevance and appropriateness to the target audience; (2) novel content development processes that iteratively combine evidence-based information with lived experience perspectives, capitalizing on multimedia (eg, videos) that the digital health space provides; (3) incorporating Web-based communities to connect end users and promote constructive engagement via access to a Web-based coach. The potential iatrogenic effects of particular exercises (eg, those of an experiential nature) within the intervention content must be considered for the target group of interest, with clinical caution messages and self-pacing encouraged to ensure end users move safely (and autonomously) through program content. Finally, sustainability models that are generalizable (eg, personnel support for guided interventions outside of an RCT context) should be considered as part of the development process.

Within the scientific context, while Web-based RCTs offer swift, unbiased randomization and allocation methods that can be fully automated, adherence and engagement with the intervention itself can be difficult to quantify. These concepts require further investigation; study designs should incorporate quantitative and qualitative assessment of adherence and engagement to move beyond automatically captured usage data and develop a richer understanding of how participants interact with Web-based interventions. These learnings can guide persuasive technologies to optimize an individual’s experience of the program. As a final method of engaging users, ORBIT seeks to build a dynamic Web-based community driven by consumers with lived experience. The ethical and scientific impact of this environment requires careful consideration; superuser roles require clear definition, seeded forum content should align with the intervention content (avoiding cross-contamination with the control condition), and ongoing supervision is required to allow forums to unfold organically while balancing scientific integrity.

Ethically, clear risk management protocols are required for Web-based self-management programs. Real-time intervention is not always possible, nor feasible, within the context of an international RCT. A guiding principle of the ORBIT project is to explicitly devolve safety and well-being to participants and their existing local care network, respecting clinical risk-management priorities and participant autonomy. While structured diagnostic interviews are central in determining inclusion criteria to RCTs, awareness of participant distress arising from such interviews requires sensitivity, careful training of clinical interviewers, and debriefing where necessary. Finally, careful consideration of legal issues surrounding the delivery of interventions claiming to have “therapeutic value” in different jurisdictions is warranted.

We offer some final insights with respect to the multifaceted skill set requirements for projects such as ORBIT that are broadly applicable to the development of any Web-based intervention. A level of technological expertise is needed to oversee the development, implementation, and evaluation of Web-based self-management programs. Expertise in this space can include knowledge of e-learning, e-communication, health informatics, basic programming skills, and awareness of technological barriers that could deter use (eg, slow internet speed and ability to watch videos), with the overall aim of ensuring that the program is innovative, engaging, feasible, and likely to be effective. As described by others [39], the ability of mental health researchers to enter the world of the Web-based program developer invites valuable opportunities to influence scoping, design, and evaluation. This offers a new skill set to mental health researchers—a new “breed of transdisciplinary experts” [39]—allowing highly innovative and clinically effective electronic mental health programs to be developed.

## Discussion

### Principal Findings

Moving forward, the digital health space offers multiple opportunities. First, Web-based self-management interventions such as ORBIT could be integrated into a stepped care approach in primary care [40]. Dissemination would occur via Primary Health networks; general practitioners are well placed to identify patients who may benefit from an evidence-based, low-intensity Web-based intervention as a first step. Stepping up or down the treatment pathway would then be determined according to patients’ needs and response to treatment. Second, hybrid treatments, where mental health practitioners and patients use Web-based programs in conjunction with face-to-face treatments (eg, within sessions together or as a way of stimulating discussions and promoting continuity of treatment outside of the treatment room), may provide alternative (or complementary) models of care. Practitioners and their patients with serious mental illness have expressed positive views about this model [41], which is currently under evaluation [42]. Third, moderated discussion forums such as those included in ORBIT may serve as stand-alone interventions; these empowered Web-based communities provide a rich, dynamic environment where consumers can exchange mental health information and receive support [43]. The peer discussion boards of the MoodSwings 2.0 Web-based self-help program for BD are currently being evaluated to clarify and maximize the benefits of Web-based discussion [44].

While clear opportunities exist in the digital health space, key challenges remain in terms of delivery and adherence. Legalities surrounding Web-based delivery of mental health interventions across different jurisdictions require urgent attention. Currently, university ethical review boards are either unaware of, or not resourced to address, legal issues arising from geographic jurisdictions other than their own [37]. For review boards to undertake appropriate vetting of clinical trials of Web-based interventions, clear identification of protective factors (eg, participants’ privacy and safety, ethical considerations, and risk issues) across jurisdictions is necessary. Transparent models for multinational internet intervention research initiatives are now needed to navigate these legalities. One component of such a model may include clearly informing participants in consenting documents that while their participation has been ethically vetted by only one institution in a given geographic and legal jurisdiction, they remain bound by legal and ethical precedents in their own geographical jurisdiction [37].

Turning to adherence with Web-based interventions, attrition is an ongoing challenge. Unlike face-to-face treatment trials where the common factors (ie, therapeutic relationship) can bolster adherence to the control condition, Web-based treatments (particularly those without guided support) pose different challenges. Research trial investigators must now make the choice to address attrition by making the engagement features of the control condition comparable to those of the preferred condition. The intriguing notion of the “therapeutic relationship” in nonguided Web-based interventions requires further exploration both in terms of attrition and treatment outcomes [45].

This discussion of insights around developing and testing a novel Web-based intervention for BD was organized in terms of three nested foci: the intervention, the science, and the ethical framework (see Figure 1). We propose that this organizing scheme is useful for future efforts in this space, particularly because it helps illuminate tensions between these three critical goals of any eTherapy project. The ORBIT project required decisions about, for example, (1) the *scientific* preference to offer a standard “no frills” control condition versus the *intervention-level* need to have best-practice engagement in the control condition; (2) the *scientific* preference to make findings as generalizable as possible versus the *ethical* need to constrain participation to minimize the risk of adverse events; and (3) the preference to have relatively unconstrained discussion on the forums to optimize impact of the *interventions* versus the *scientific* goal of stimulus control and the *ethical* goal of minimizing triggering statements for other participants. The ultimate development or testing of any Web-based intervention rests heavily on the contextualized, procedural positions taken on these multifaceted issues, reminding us again of the substantial gap between the simple content of any psychological intervention and its instantiation in a Web-based delivery platform.

### Limitations

This paper aimed to support future investigation of Web-based interventions for mental health conditions by describing the minutiae of the clinical, scientific, and ethical decision making underpinning one particular Web-based trial. A potential limitation of the paper, then, is the extent to which insights from the ORBIT trial are specific to this population (patients with BD), this intervention (brief novel self-help strategies), or this outcome variable (quality of life). We encourage readers to be attentive to these particulars as they draw generalizations for their own innovative Web-based interventions.

### Conclusion

Technology allows for highly interactive and engaging programs that empower participants to manage their mental health. This departure from the care model can prompt clinician insecurity (treatments that work are arguably provocative and, therefore, potentially destabilizing); however, it should not be a barrier to offering the intervention to consumers. As overviewed by others [12,17], there is evidence that self-management is effective in BD, with those on the more severe end of the spectrum still able to learn to self-manage and take control of their lives. While there are challenges to be aware of, guided self-management programs such as those offered by the ORBIT project that are specifically developed for Web-based delivery provide highly accessible, engaging, and potentially provocative treatments for chronically ill populations who may otherwise have never engaged with treatment. Key questions about engagement, effectiveness, and cost-effectiveness will be answered by the ORBIT project over the next 18 months.

## Abbreviations

BD: bipolar disorder
CAG: consumer advisory group
HREC: Human Research Ethics Committee
ORBIT: Online, Recovery-Oriented Bipolar Individualised Tool
RCT: randomized controlled trial

## References

[1] Cuijpers P, van Straten A, Warmerdam L, van Rooy MJ (2010). Recruiting participants for interventions to prevent the onset of depressive disorders: possible ways to increase participation rates. BMC Health Serv Res.

[2] Donker T, Blankers M, Hedman E, Lótsson B, Petrie K, Christensen H (2015). Economic evaluations of Internet interventions for mental health: a systematic review. Psychol Med.

[3] Titov N, Dear B, Ali S, Zou J, Lorian C, Johnston L, Terides M, Kayrouz R, Klein B, Gandy M, Fogliati Vincent J (2015). Clinical and cost-effectiveness of therapist-guided internet-delivered cognitive behavior therapy for older adults with symptoms of depression: a randomized controlled trial. Behav Ther.

[4] Lal S, Adair C (2014). E-mental health: a rapid review of the literature. Psychiatr Serv.

[5] Carlbring P, Andersson G, Cuijpers P, Riper H, Hedman-Lagerlöf Erik (2018). Internet-based vs. face-to-face cognitive behavior therapy for psychiatric and somatic disorders: an updated systematic review and meta-analysis. Cogn Behav Ther.

[6] Andersson G, Cuijpers P, Carlbring P, Riper H, Hedman E (2014). Guided Internet-based vs. face-to-face cognitive behavior therapy for psychiatric and somatic disorders: a systematic review and meta-analysis. World Psychiatry.

[7] Andrews G, Basu A, Cuijpers P, Craske M, McEvoy P, English C, Newby J (2018). Computer therapy for the anxiety and depression disorders is effective, acceptable and practical health care: An updated meta-analysis. J Anxiety Disord.

[8] Murray G, Leitan N, Berk M, Thomas N, Michalak E, Berk L, Johnson S, Jones S, Perich T, Allen N, Kyrios Michael (2015). Online mindfulness-based intervention for late-stage bipolar disorder: pilot evidence for feasibility and effectiveness. J Affect Disord.

[9] Fletcher K, Foley Fiona, Thomas N, Michalak E, Berk L, Berk M, Bowe S, Cotton S, Engel L, Johnson S, Jones Steven, Kyrios Michael, Lapsley Sara, Mihalopoulos Cathrine, Perich Tania, Murray Greg (2018). Web-based intervention to improve quality of life in late stage bipolar disorder (ORBIT): randomised controlled trial protocol. BMC Psychiatry.

[10] Susman JL (2010). Improving outcomes in patients with bipolar disorder through establishing an effective treatment team. Prim Care Companion J Clin Psychiatry.

[11] Bauer MS, Biswas K, Kilbourne AM (2009). Enhancing multiyear guideline concordance for bipolar disorder through collaborative care. Am J Psychiatry.

[12] Leitan N, Michalak E, Berk L, Berk M, Murray G (2015). Optimizing delivery of recovery-oriented online self-management strategies for bipolar disorder: a review. Bipolar Disord.

[13] Christensen H, Petrie K (2013). State of the e-mental health field in Australia: where are we now?. Aust N Z J Psychiatry.

[14] Case AD, Byrd R, Claggett E, DeVeaux S, Perkins R, Huang C, Sernyak MJ, Steiner JL, Cole R, LaPaglia DM, Bailey M, Buchanan C, Johnson A, Kaufman JS (2014). Stakeholders' perspectives on community-based participatory research to enhance mental health services. Am J Community Psychol.

[15] Thomas N, Farhall J, Foley F, Leitan ND, Villagonzalo K-A, Ladd E, Nunan C, Farnan S, Frankish R, Smark T, Rossell Susan L, Sterling Leon, Murray Greg, Castle David Jonathon, Kyrios Michael (2016). Promoting Personal Recovery in People with Persisting Psychotic Disorders: Development and Pilot Study of a Novel Digital Intervention. Front Psychiatry.

[16] Thomas N, McLeod B, Jones N, Abbott J (2015). Developing Internet interventions to target the individual impact of stigma in health conditions. Internet Interventions.

[17] Todd N, Solis-Trapala I, Jones S, Lobban F (2012). An online randomised controlled trial to assess the feasibility, acceptability and potential effectiveness of 'Living with Bipolar': a web-based self-management intervention for bipolar disorder: trial design and protocol. Contemp Clin Trials.

[18] Wilhelmsen M, Lillevoll K, Risør Mette Bech, Høifødt Ragnhild, Johansen M, Waterloo K, Eisemann M, Kolstrup N (2013). Motivation to persist with internet-based cognitive behavioural treatment using blended care: a qualitative study. BMC Psychiatry.

[19] Murray G, Leitan N, Thomas N, Michalak E, Johnson S, Jones S, Perich T, Berk L, Berk M (2017). Towards recovery-oriented psychosocial interventions for bipolar disorder: Quality of life outcomes, stage-sensitive treatments, and mindfulness mechanisms. Clin Psychol Rev.

[20] Fives A, Rusell D, Kearns N, Lyons R, Eaton P, Canavan J, Devaney C, O'Brien A (2013). The role of random allocation in randomized controlled trials: distinguishing selection bias from baseline imbalance. J MultiDiscipl Eval.

[21] Murray E (2012). Web-based interventions for behavior change and self-management: potential, pitfalls, and progress. Med 2 0.

[22] Eysenbach G (2005). The law of attrition. J Med Internet Res.

[23] Donkin L, Christensen H, Naismith S, Neal B, Hickie I, Glozier Nick (2011). A systematic review of the impact of adherence on the effectiveness of e-therapies. J Med Internet Res.

[24] Proudfoot J, Klein B, Barak A, Carlbring P, Cuijpers P, Lange A, Ritterband L, Andersson G (2011). Establishing guidelines for executing and reporting Internet intervention research. Cogn Behav Ther.

[25] Doherty G, Coyle D, Sharry J (2012). Engagement with online mental health interventions: an exploratory clinical study of a treatment for depression. https://dl.acm.org/citation.cfm?id=2208602.

[26] Pachankis JE, Lelutiu-Weinberger C, Golub SA, Parsons JT (2013). Developing an online health intervention for young gay and bisexual men. AIDS Behav.

[27] Graffigna G, Barello S, Bonanomi A, Lozza E (2015). Measuring patient engagement: development and psychometric properties of the Patient Health Engagement (PHE) Scale. Front Psychol.

[28] Tetley A, Jinks M, Huband N, Howells K (2011). A systematic review of measures of therapeutic engagement in psychosocial and psychological treatment. J Clin Psychol.

[29] Kelders S, Kok R, Ossebaard H, Van Gemert-Pijnen JEWC (2012). Persuasive system design does matter: a systematic review of adherence to web-based interventions. J Med Internet Res.

[30] Perski O, Blandford A, West R, Michie S (2017). Conceptualising engagement with digital behaviour change interventions: a systematic review using principles from critical interpretive synthesis. Transl Behav Med.

[31] Murray G, Leitan N, Michalak E, Berk L, Berk M (2015). Supporting self-management in bipolar disorder online: a review.

[32] Todd N, Jones S, Hart A, Lobban F (2014). A web-based self-management intervention for Bipolar Disorder 'living with bipolar': a feasibility randomised controlled trial. J Affect Disord.

[33] Lauder S, Chester A, Castle D, Dodd S, Berk L, Klein B, Austin D, Gilbert M, Chamberlain JA, Murray G, White C, Piterman L, Berk M (2013). Development of an online intervention for bipolar disorder. www.moodswings.net.au. Psychol Health Med.

[34] Todd NJ, Jones SH, Lobban FA (2013). What do service users with bipolar disorder want from a web-based self-management intervention? A qualitative focus group study. Clin Psychol Psychother.

[35] Naslund John A, Marsch Lisa A, McHugo Gregory J, Bartels Stephen J (2015). Emerging mHealth and eHealth interventions for serious mental illness: a review of the literature. J Ment Health.

[36] Lauder S, Chester A, Castle D, Dodd S, Gliddon E, Berk L, Chamberlain J, Klein B, Gilbert M, Austin D, Berk Michael (2015). A randomized head to head trial of MoodSwings.net.au: an Internet based self-help program for bipolar disorder. J Affect Disord.

[37] Cosgrove V, Gliddon E, Berk L, Grimm D, Lauder S, Dodd S, Berk M, Suppes T (2017). Online ethics: where will the interface of mental health and the internet lead us?. Int J Bipolar Disord.

[38] Sheehan D V, Lecrubier Y, Sheehan K H, Amorim P, Janavs J, Weiller E, Hergueta T, Baker R, Dunbar G C (1998). The Mini-International Neuropsychiatric Interview (M.I.N.I.): the development and validation of a structured diagnostic psychiatric interview for DSM-IV and ICD-10. J Clin Psychiatry.

[39] Pagliari C (2007). Design and evaluation in eHealth: challenges and implications for an interdisciplinary field. J Med Internet Res.

[40] Ebert Dd, Van Daele T, Nordgreen T, Karekla M, Compare A, Zarbo C, Brugnera A, Øverland S, Trebbi G, Jensen Kl, Kaehlke F, Baumeister H (2018). Internet- and Mobile-Based Psychological Interventions: Applications, Efficacy, and Potential for Improving Mental Health. European Psychologist.

[41] Thomas N, Foley F, Lindblom K, Lee S (2017). Are people with severe mental illness ready for online interventions? Access and use of the Internet in Australian mental health service users. Australas Psychiatry.

[42] Thomas N, Farhall J, Foley F, Rossell SL, Castle D, Ladd E, Meyer D, Mihalopoulos C, Leitan N, Nunan C, Frankish R, Smark T, Farnan S, McLeod B, Sterling L, Murray G, Fossey E, Brophy L, Kyrios M (2016). Randomised controlled trial of a digitally assisted low intensity intervention to promote personal recovery in persisting psychosis: SMART-Therapy study protocol. BMC Psychiatry.

[43] Harding C, Chung H (2016). Behavioral health support and online peer communities: international experiences. Mhealth.

[44] Gliddon E, Lauder S, Berk L, Cosgrove V, Grimm D, Dodd S, Suppes T, Berk M (2015). Evaluating discussion board engagement in the MoodSwings online self-help program for bipolar disorder: protocol for an observational prospective cohort study. BMC Psychiatry.

[45] Cavanagh K, Millings A (2013). (Inter)personal Computing: The Role of the Therapeutic Relationship in E-mental Health. J Contemp Psychother.

